# Surgical Excision Followed by Low Dose Rate Radiotherapy in the Management of Resistant Keloids

**Published:** 2013-06

**Authors:** Ali Akbar Mohammadi, Mohammad Mohammadian Panah, Mohammad Reza Pakyari, Raziyeh Tavakol, Iman Ahrary, Seyed Morteza Seyed Jafari, Maryam Sharifian

**Affiliations:** 1 Shiraz Burn Research Center, Division of Plastic and Reconstructive Surgery, Department of Surgery, Shiraz University of Medical Sciences, Shiraz, Iran;; 2 Department of Radiation Oncology, Shiraz University of Medical Sciences, Shiraz, Iran

**Keywords:** Surgical excision, Radiotherapy, Keloid

## Abstract

**BACKGROUND:**

Depending on the cause, 40-90% of every deep dermis insult ends up in scar formation. Several modalities have been suggested as a treatment but a high rate of recurrence is reported in most of those interventions. High dose radiotherapy has been shown to be effective in reducing the recurrence rate. This study tried to determine the effectiveness of low dose rate radiotherapy following surgical excision in treating resistant keloids.

**METHODS:**

Between January 2008 and April 2011, seventeen patients (mostly burn patients) with 26 keloids went through surgical resection followed by radiotherapy. A total dose of 15 Gy in 5 fractions was administered to the areas of scar formation.

**RESULTS:**

All patients were followed for at least 11 months (mostly for 20 months). No recurrence occurred. There was no complication or adverse effect.

**CONCLUSION:**

Surgical excision followed by low dose postoperative radiotherapy was an efficient treatment for keloids that were resistant to many other modalities**.**

## INTRODUCTION

Every year, over a hundred million patients in western countries are noticed with scars as a result of ninety million elective or post trauma surgeries.^1^ The prevalence of hypertrophic scar and keloids among Caucasians is really unknown. Some previous reviews implied that hypertrophic scar ranged from 40 to 70% following surgeries and 91% following burn injuries.^2^ The prevalence is even higher among Asians and Africans. These numbers indicate that the scar formation remains a major problem and the most common complication is deep dermis insult.

Any sort of insult to the deeper layers of dermis including trauma, ear piercing, burn injuries may develop keloids or excessive scar formation. Several therapeutic strategies have been suggested for prevention or treatment of keloids and hypertrophic scar formation but a few of these strategies have been supported in prospective surveys with suitable control groups.^3^ Durani and Bayat published a review on evaluation of proposed therapeutic techniques over the last twenty five years. They concluded that “high quality research in evaluating keloid therapy is still lacking”.^4^

Although developing hypertrophic scars and keloids are seen mostly in post burn injuries (91%), there are not so much studies, investigating scar and keloid management in burn injuries. In this study, we tried to include a greater percentage of patients with burn injuries as a cause of scar formation. Surgical treatment of keloids causes a high recurrence rate (up to 80% of the cases).^5^ This point has led to use adjuvant therapies, as complementary treatment, to reduce the rate of recurrence, topical silicone,^6^ laser excision,^7^ steroid injection,^8^ cryosurgery,^9^ and post-operative radiation.^10^^,^^11^ Post-operative radiation has been proved to be efficient in preventing keloid formation and recurrence. In the present trial, the authors tried to outline the results of their experience in utilizing low doses of post-operative radiation as an adjuvant treatment, in patients with resistant keloids.

## MATERIALS AND METHODS

From January 2008 to April 2011, seventeen patients with 26 keloids went through surgical resection followed by radiotherapy. The method of case selection and matching was easy sampling (every patient who came to our office and met the inclusion criteria without any exclusion criteria were enrolled). The Ethics Committee of Shiraz University of Medical Sciences approved this study with the requirement of patient informed consent.

Every patient between 20 and 60 years old with a clinically confirmation for keloid formation and the history of at least 3 previously failed interventions were included. Our exclusion criteria were pregnancy, collagen vascular disorders, diabetes, and other hormonal impairments. We also excluded those patients who refused to sign the written informed consent.

At the very first stage of the study, the scars were examined regarding to their poligonality, depth, color, and size. These examinations were supposed to differentiate the malignant scars (scars with bizarre shape, color and depth and so forth increase the probability of malignant scars) from the benign ones. Vancouver scar scale was used for scoring the keloid concerning vascularity, pigmentation, pliability and height. Accordingly, the patients were divided into 4 groups regarding to the severity of the scars (Table 1).

**Table 1:** The patients groups according to severity of the scar

**Groups**	**Scar Scale according to Vancouver score**
0 (Normal)	0
1 (Mild)	1-4
2 (Moderate)	5-8
3 ( Severe )	9-13

After scar scoring and taking written informed consent, surgical excision was done within few days. By the next 24 hour after total surgical resection of keloids, radiotherapy course was started. The whole radiotherapy course was consisted of 5 time’s daily fractions with 3 Gray radiation with superficial X-ray made up of 120 KW energy photons. Radiation zone was the area of resected scar with one centimeter margin. Thus, the cumulative radiation in the entire course of study was 15 Gray, consisted of superficial X-ray photon, divided into 5 daily fractions.

All the patients were re-examined by a radio-oncologist and the researchers. The times of examinations were just after completion of the radiation course, every 2 weeks in the first month, every 1 month for three month and six months after that and all patients were reexamined at the end of the study to determine their final Vancouver Scar Score. In every follow up visit, the patients were examined for developing any sort of adverse effects or complication. The Vancouver Scar scores of the patients before incision were compared with the scores of the patients in the final session to see the effect of radiation course.

All these data were gathered within 36 months of consecutive study. Then the data were transported to SPSS software (Version 13, Chicago, IL, USA) for analysis. The frequency tests, cross tabulation, and the one way ANOVA test were used for analysis. We also explored our data for advanced descriptive analysis. A p value less than 0.05 was considered statistically significant.

## RESULTS

Seventeen patients were enrolled in this study. There were eight (48%) males and nine (52%) females. The mean age of the patients at treatment was 27.17 years (SD: 7.10) and the mean duration of the disease was 4.5 years (SD: 3.0). Previous therapeutic interventions which were done for the patients are listed in Table 2. The mean period of follow up was 16.35 months with minimum and maximum period of 9 and 36 months (SD: 7.11). 

**Table 2:** Previous therapeutic interventions

**Therapeutic interventions **	**Number**
Surgery	37
Corticosteroids	54
Cryosurgery	26
Laser	6

**Fig. 1:**
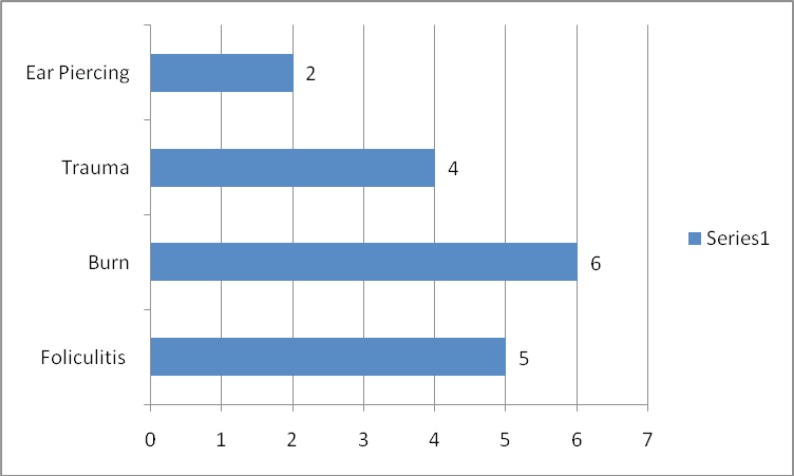
Primary cause of the skin injury

**Fig 2:**
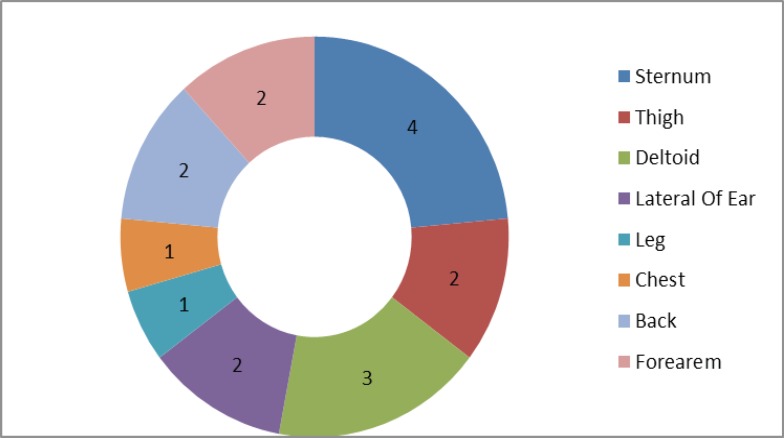
The site of keloid formation

The primary cause of skin injury was shown in Figure 1 and the site of keloid formation was shown in Figure 2.

**Fig. 3:**
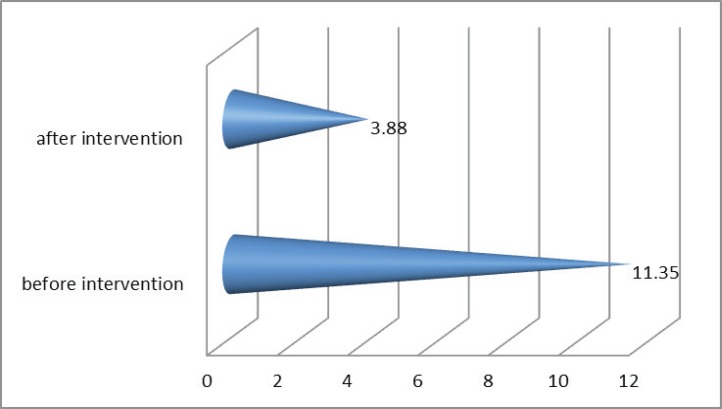
Vancouver scale before and after intervention

**Fig. 4:**
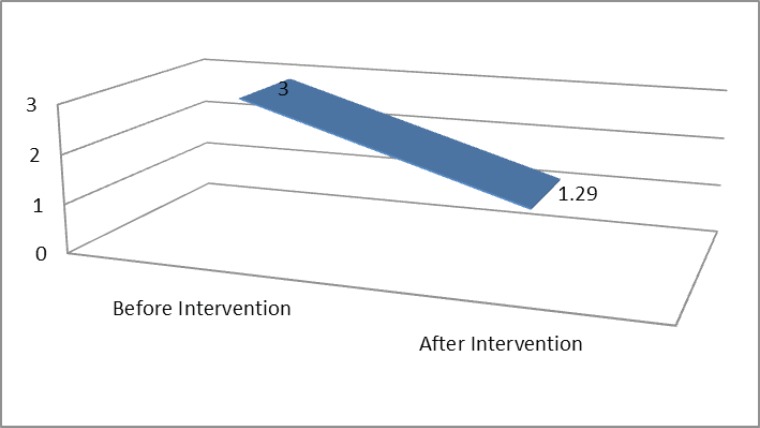
Severity score before and after intervention

The mean Vancouver Scar Scale before intervention and one year after radiotherapy was 11.35 (SD: 0.99) and 3.88 (SD: 1.69) respectively, (Figure 3). And at last the severity score according to the results were significantly decreased after the intervention (p. value < 0.005) (Figure 4). 

## DISCUSSION

As it was mentioned before, keloids are commonly refractory to many of current treatments and although many types of therapeutic methods have been suggested for this condition, there is not a universally accepted method.^4^ Fifteen out of 17 patients of this study were previously treated by surgical excision and intra-lesional injections of corticosteroid with almost 100% of recurrence at the time going to our office. This point revealed the important fact that, although surgical resection followed by corticosteroid injections reduced the rate of recurrence in comparison to surgical excision, but it still had a significant recurrence in a long time period. 

Several surveys have studied the rate of recurrence in surgical excision as a monotherapy in comparison with surgical therapy combined with adjuvant therapies such as steroid injections and post-operative radiotherapy. Almost all of them concluded that the former methods had a significant higher rate of recurrence than the latest one. The differences in decreasing rate of recurrence in different studies might be dependent on factors such as the dose of radiation, the number of sessions of radiation and the interval between performing surgery and starting radiotherapy.^14^^-^^16^

In this study, radiotherapy soon after surgery caused a significant decrease in the Vancouver Scale Score of the scar. During 16 months follow up, we did not detect any case with recurrence or post intervention complications. These results demonstrated that radiotherapy especially in the large and recurrence cases was an effective method of treatment. Although the total radiation dose in this study (15 Gy in five daily fractions) was half of the dose recommended in Kal et al. study, similar to them, we suggested radiation soon after surgical excision for achieving the best outcome.^17^

Safety and effectiveness of the method used in this study was the same as what mentioned in many other previous surveys.^14^^,^^18^ The cosmetic results in the outcome of the patients were acceptable similar to the study of Recalcati *et al.*^19^ The rate of recurrence was very low and minimal rate of complications was seen in follow up sessions. The probable differences in clinical and histological features of keloids happened by different causes are not appraised in any previous study. The most frequent cause of primary skin injury in this study was burn. Studying burns as major cause of hypertrophic scars and keloid formation aside from other reasons is lacking in previous surveys. 

The results of this study might reveal a better view of keloid formation and its efficient treatment in the burn patients. Surgical excision followed by postoperative radiotherapy was an effective way for the treatment of keloids which were resistant to other modalities. No recurrence or complication occurred after the intervention. Although it seems safe and efficient, further surveys with more patients should be performed to confirm the efficacy of low dose rate radiotherapy in large populations. 

## Footenote

Please cite this paper as:

Mohammadi AA, Mohammadian Panah M, Pakyari MR, Tavakol R, Ahrary I, Seyed Jafari SM, Sharifian M. Surgical Excision Followed by Low Dose Rate Radiotherapy in the Management of Resistant Keloids. World J Plast Surg 2013;2(2):81-86.
